# Supplementary figures and images for: Naturally occurring variation in a cytochrome P450 modifies thiabendazole responses independently of beta-tubulin
Source: PLoS Pathog. 2025 Jan 14;21(1):e1012602. doi: 10.1371/journal.ppat.1012602 (PMC11771912; doi:10.1371/journal.ppat.1012602)

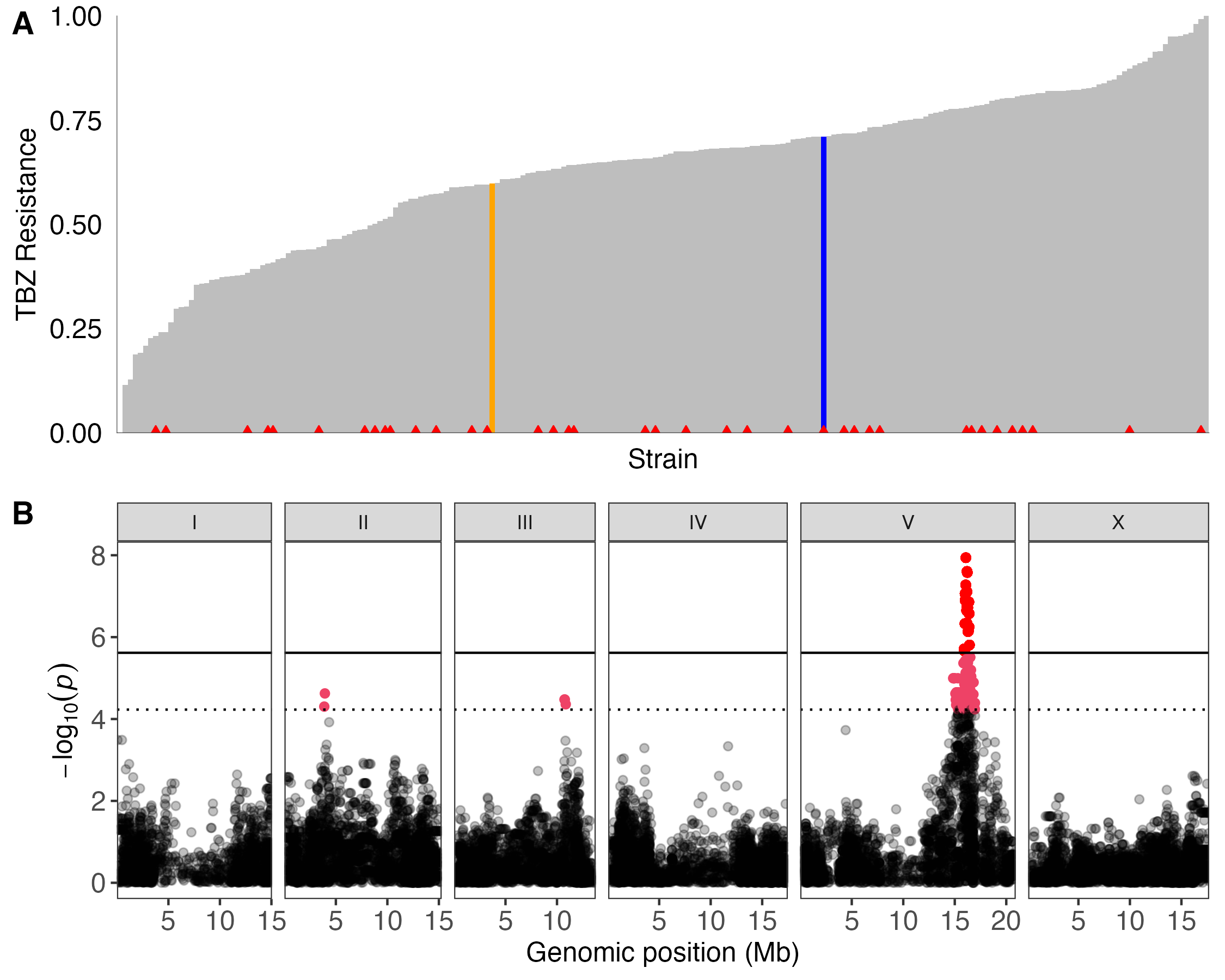

Supplement: S1 Fig — (A) Distribution of normalized TBZ response after regression using ben-1 variation as a covariate is shown ordered from most susceptible to most resistant. The N2 and CB4856 strains are colored orange and blue, respectively. Strains with variation in ben-1 have a red triangle at the base of the bar for that strain. (B) Genome-wide association mapping results for animal length following ben-1 regression are shown. The genomic position is shown on the x-axis, and -log10(p) values are shown on the y-axis for each SNV. SNVs are colored pink if they pass the Eigen threshold (dashed horizontal line) or red if they pass the Bonferroni significance threshold (horizontal line). (TIF) [file ppat.1012602.s020.tif]

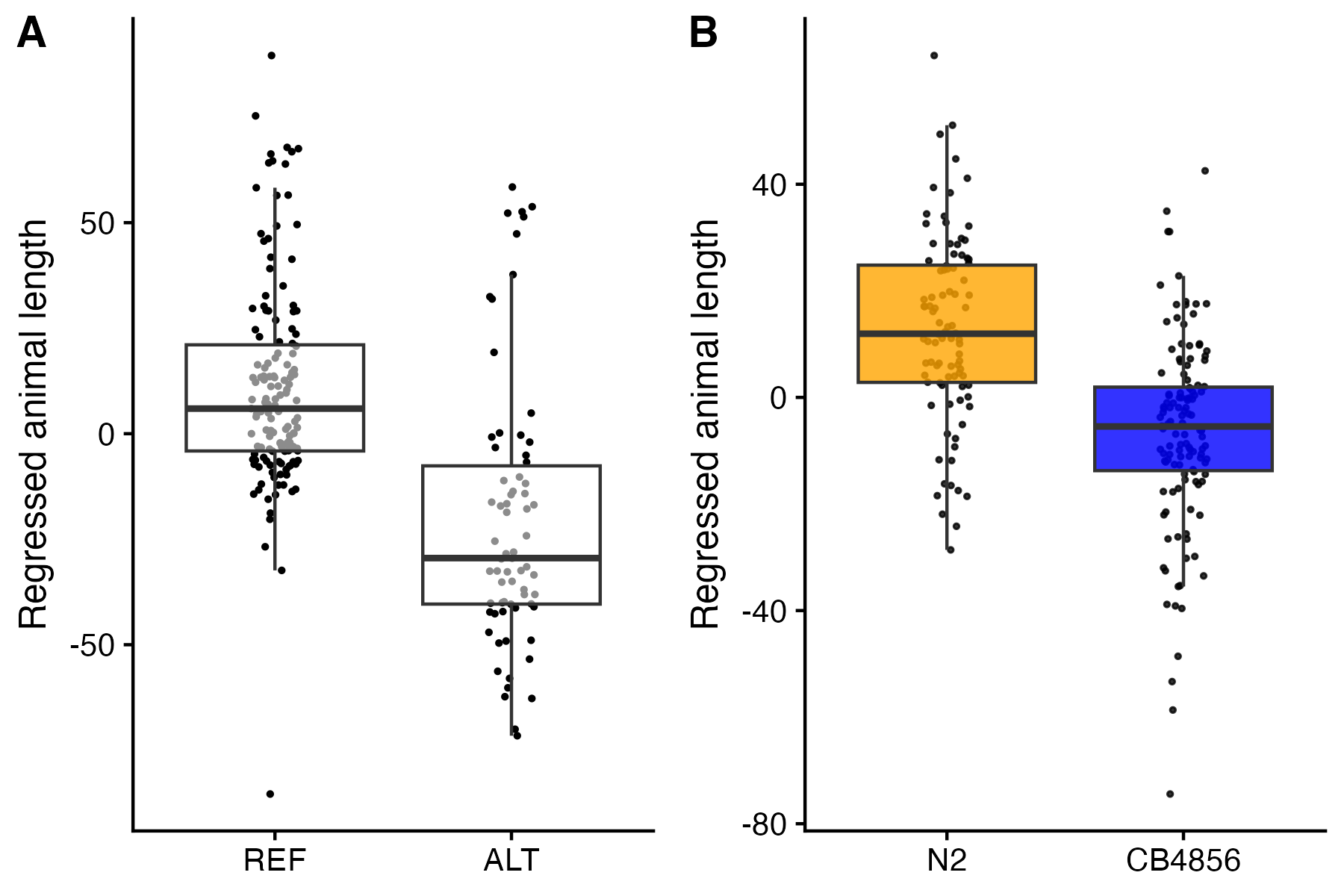

Supplement: S2 Fig — (A) Regressed animal length (mean.TOF) values in response to TBZ treatment in a genome-wide association study are shown on the y-axis. The x-axis denotes if a strain has the lysine (REF) or glutamate (ALT) alleles at the peak marker. Each point represents a strain’s normalized response from multiple replicates. (B) For the QTL discovered in the linkage mapping experiment, the regressed animal length (mean.TOF) is shown on the y-axis, and the allele of the tested recombinant strain is shown on the x-axis. Data are shown as box plots with the median as a solid horizontal line, the top and bottom of the box representing the 75th and 25th quartiles, respectively. (TIF) [file ppat.1012602.s021.tif]

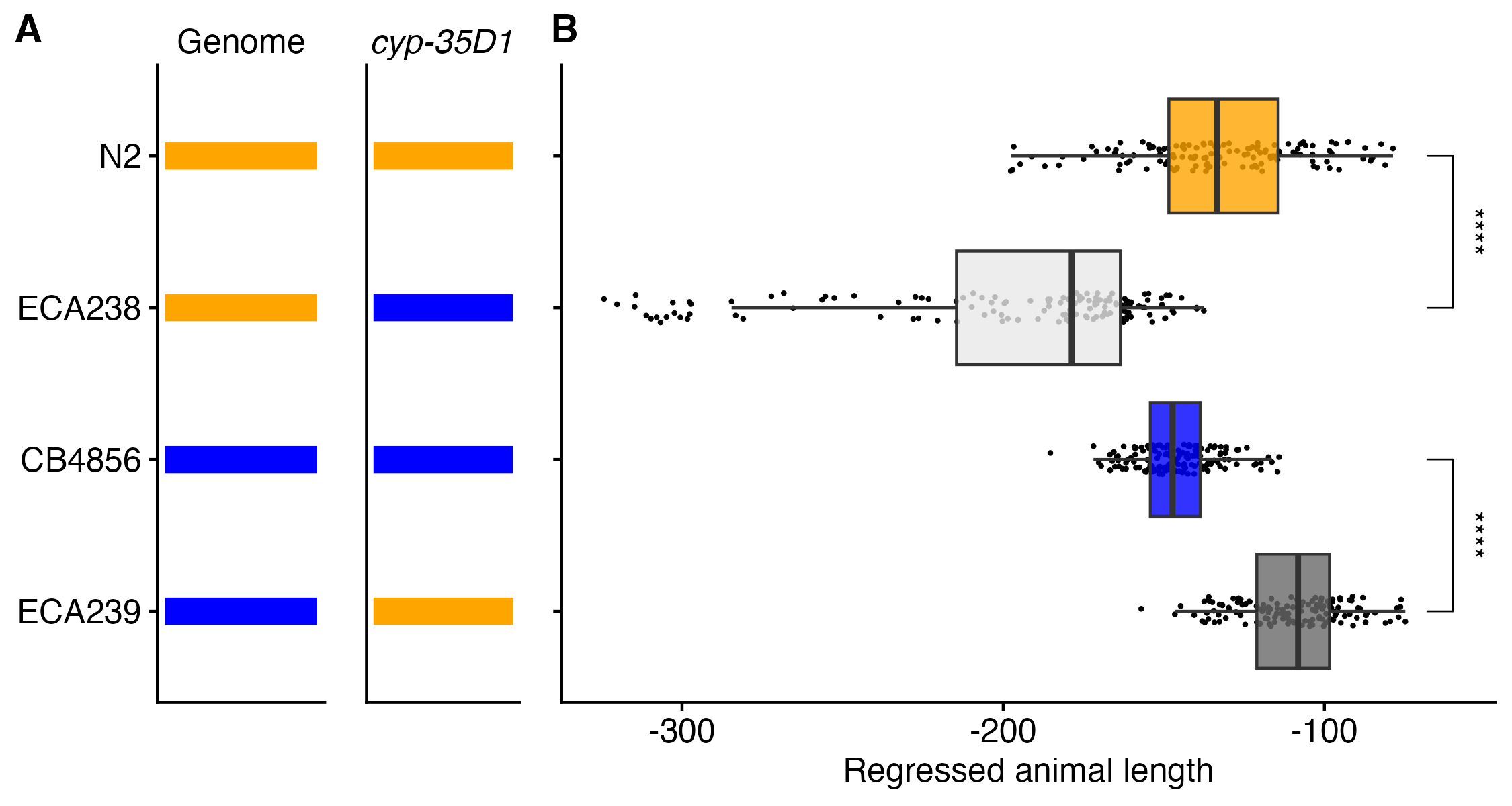

Supplement: S3 Fig — The strain tested is shown on the y-axis. (A) Chromosome V of each strain with the genotype at markers across chromosome V is shown. The x-axis shows the genomic position across the chromosome. A red line denotes the location of the QTL on chromosome V. The genomic background of the cyp-35D1 locus is shown orange for N2 and blue for CB4856. (C) Regressed animal length (mean.TOF) in response to TBZ treatment is shown on the x-axis. The ECA238 strain is a near-isogenic line in the N2 genetic background with a small introgression of the CB4856 genome around the identified chromosome V QTL from linkage mapping. The ECA239 strain is a near-isogenic line in the CB4856 background with a small introgression of the N2 genome around the identified QTL from linkage mapping. Data are shown as Tukey box plots with the median as a solid vertical line, the right and left of the box representing the 75th and 25th quartiles, respectively. Statistical significance in comparison to the genomic background strain is shown to the right (p < 0.0001 = ****, Tukey HSD). (TIF) [file ppat.1012602.s022.tif]

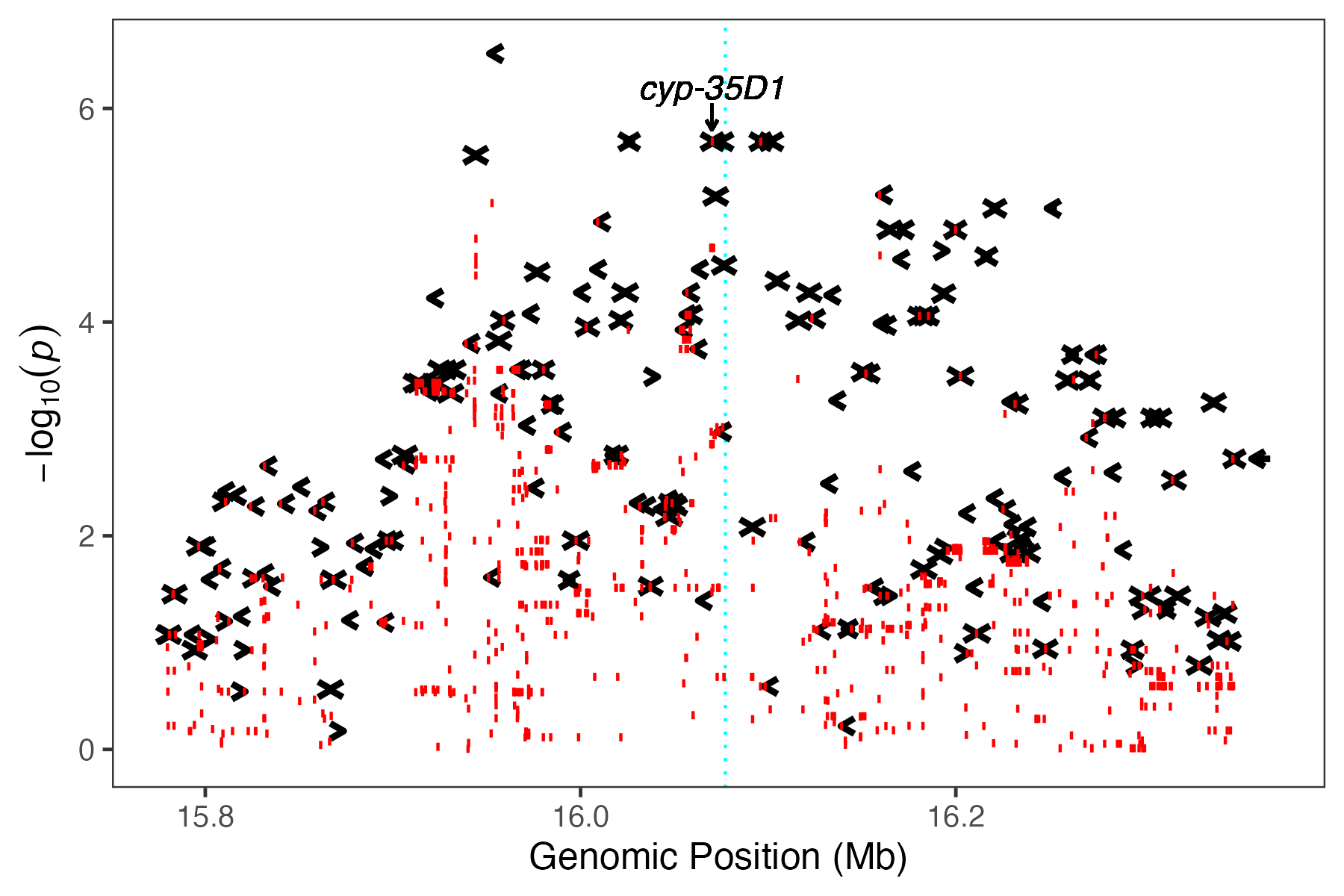

Supplement: S4 Fig — Fine mapping of the QTL region on chromosome V is displayed for (A) non-ben-1-regressed data. Each gray bar represents a gene in the region of interest. Red bars indicate high-impact variants in the region, black arrows indicate the direction of gene transcription, and the association between TBZ response and the variant is shown on the y-axis. The location of cyp-35D1 is shown with a label and an arrow pointing to the gene and variant location (TIF) [file ppat.1012602.s023.tif]

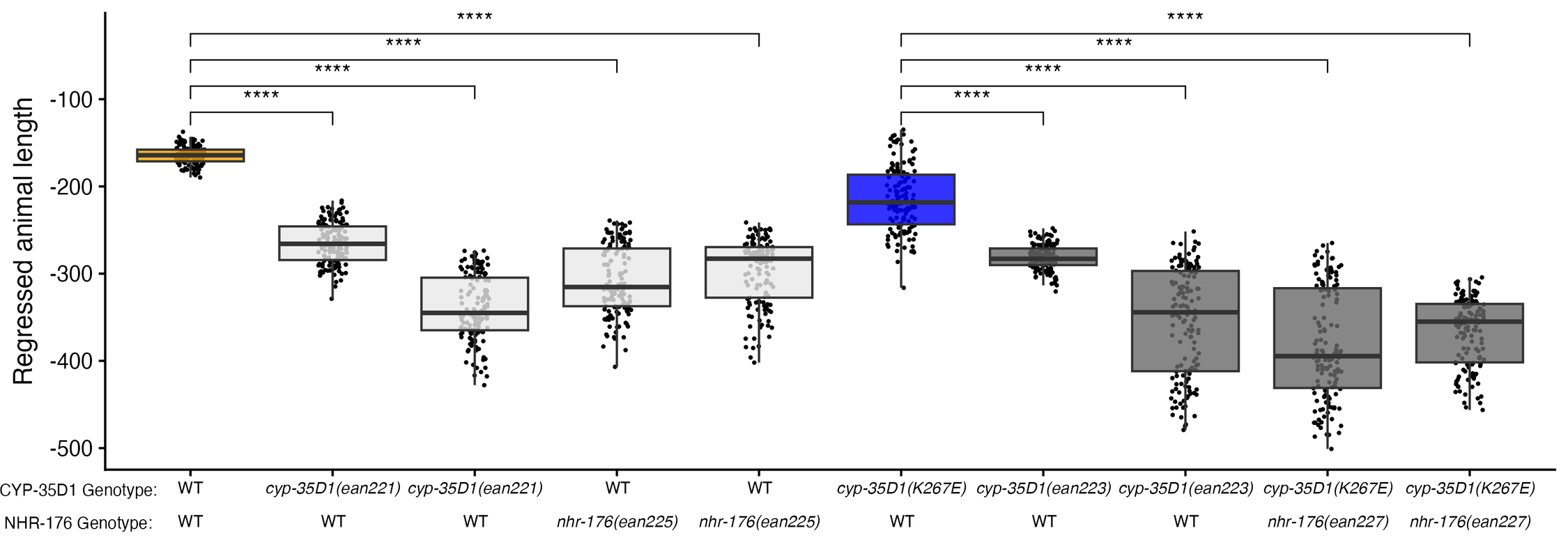

Supplement: S5 Fig — The strain cyp-35D1 genotype (top) and nhr-176 genotype (bottom) are shown on the x-axis, with WT representing the wild type for each gene. N2 (orange) and CB4856 (blue) are shown with comparisons to strains within the same genetic background. Data from both independent edits for each edit are shown. Regressed median animal length values of response to TBZ are shown on the y-axis Each point represents a well that contains ~30 animals after 48 hours of exposure to TBZ. Data are shown as box plots with the median as a solid horizontal line, the top and bottom of the box representing the 75th and 25th quartiles, respectively. The top and bottom whiskers are extended to the maximum point that is within 1.5 interquartile range from the 75th and 25th quartiles, respectively. Statistical significance in comparison to the genomic background strain is shown above each strain (p < 0.0001 = ****, Tukey HSD). (TIF) [file ppat.1012602.s024.tif]

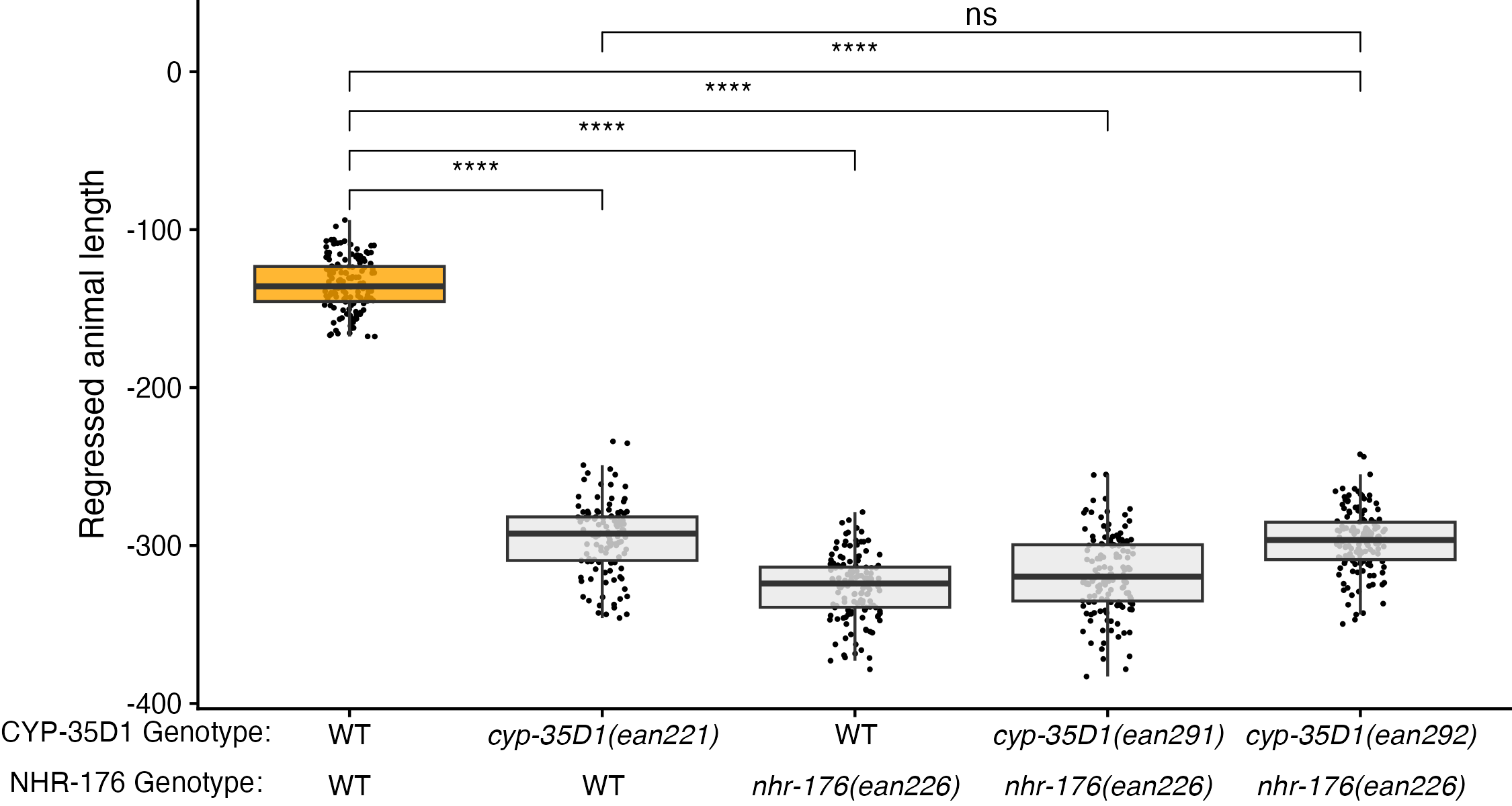

Supplement: S6 Fig — The strain cyp-35D1 genotype (top) and nhr-176 genotype (bottom) are shown on the x-axis with WT representing the wild type for each gene. N2 (orange) and CB4856 (blue) are shown for comparisons with genetic backgrounds. Regressed median animal length values of response to TBZ are shown on the y-axis Each point represents a well that contains ~30 animals after 48 hours of exposure to TBZ. Data are shown as box plots with the median as a solid horizontal line, the top and bottom of the box representing the 75th and 25th quartiles, respectively. The top and bottom whiskers are extended to the maximum point that is within 1.5 interquartile range from the 75th and 25th quartiles, respectively. Statistical significance in comparison to the genomic background strain is shown above each strain (p < 0.0001 = ****, Tukey HSD). (TIF) [file ppat.1012602.s025.tif]

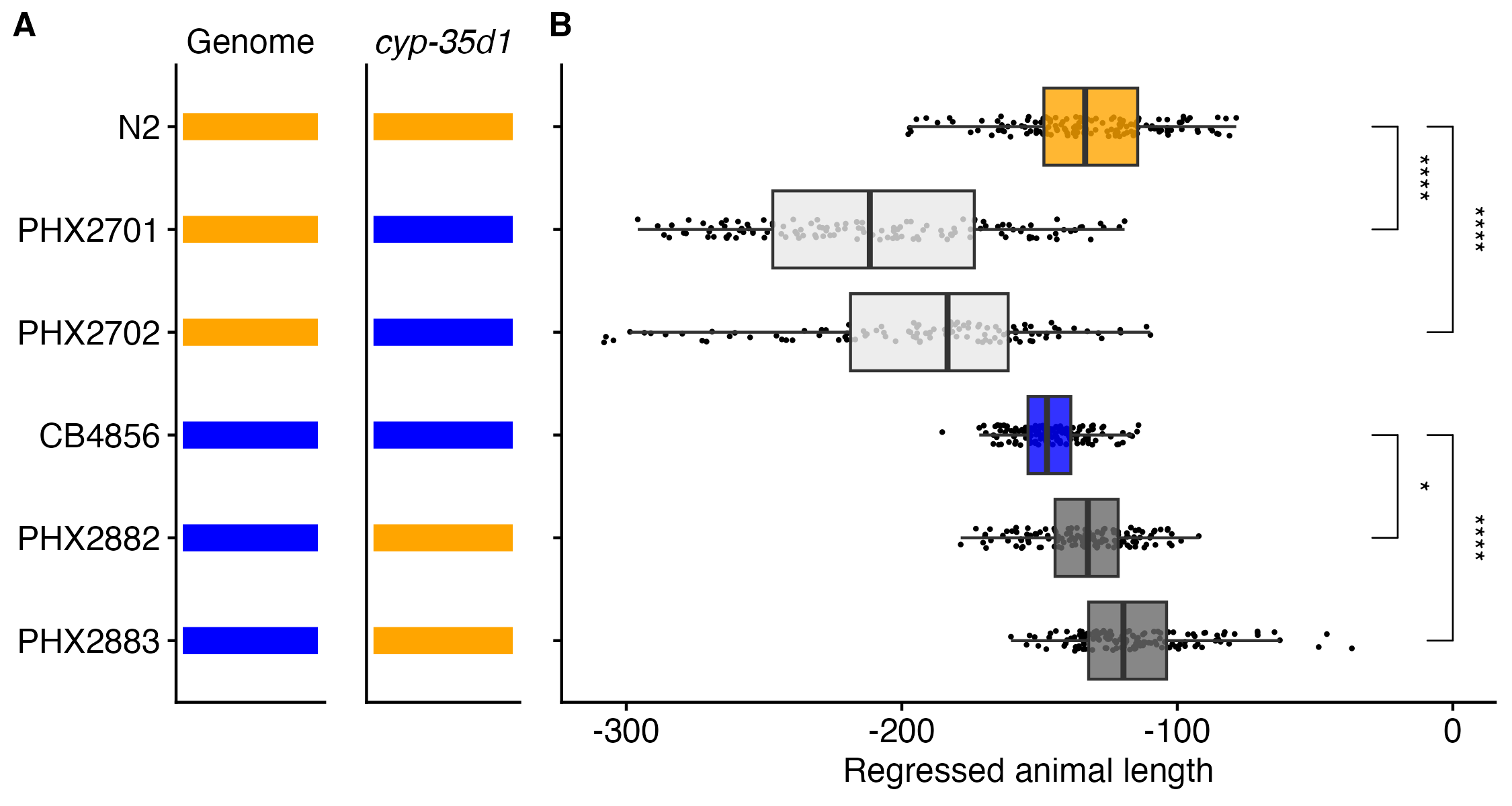

Supplement: S7 Fig — The strain name is displayed on the y-axis. (A) The genomic background of the tested strain is shown as orange or blue for N2 and blue CB4856, respectively. The CYP-35D1 allele for the strain tested is shown as orange for K267 and blue for E267. (C) Regressed animal length (mean.TOF) values of response to TBZ are shown on the x-axis. Each point represents a well that contains hundreds of animals following 96 hours of TBZ treatment. Data are shown as box plots with the median as a solid vertical line, the right and left of the box representing the 75th and 25th quartiles, respectively. The left and right whiskers are extended to the maximum point that is within 1.5 interquartile range from the 25th and 75th quartiles, respectively. Statistical significance in comparison to the genomic background strain is shown to the right of each strain; N2 and CB4856 are also significantly different (p<0.05 = *, p < 0.0001 = ****, Tukey HSD). (TIF) [file ppat.1012602.s026.tif]

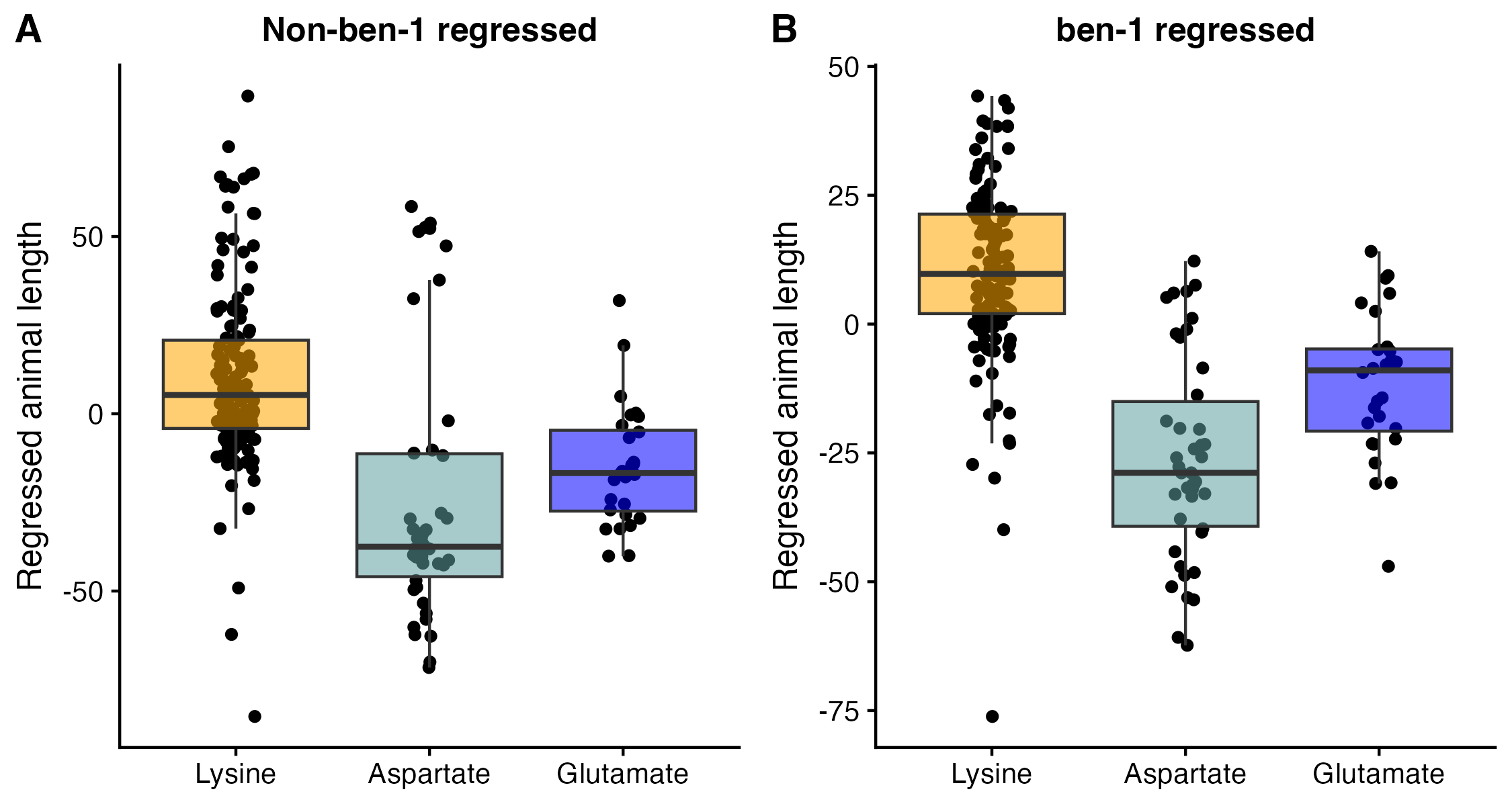

Supplement: S8 Fig — Regressed mean animal length values of responses to TBZ, both non-regressed by ben-1 (A) and regressed by ben-1 (B), are shown on the y-axis. Each point represents the regressed mean animal length value from hundreds of animals in a single well. The x-axis shows the CYP-35D1 allele. Data are shown as box plots with the median as a solid horizontal line, the top and bottom of the box representing the 75th and 25th quartiles, respectively. The top and bottom whiskers are extended to the maximum point that is within 1.5 interquartile range from the 75th and 25th quartiles, respectively. (TIF) [file ppat.1012602.s027.tif]

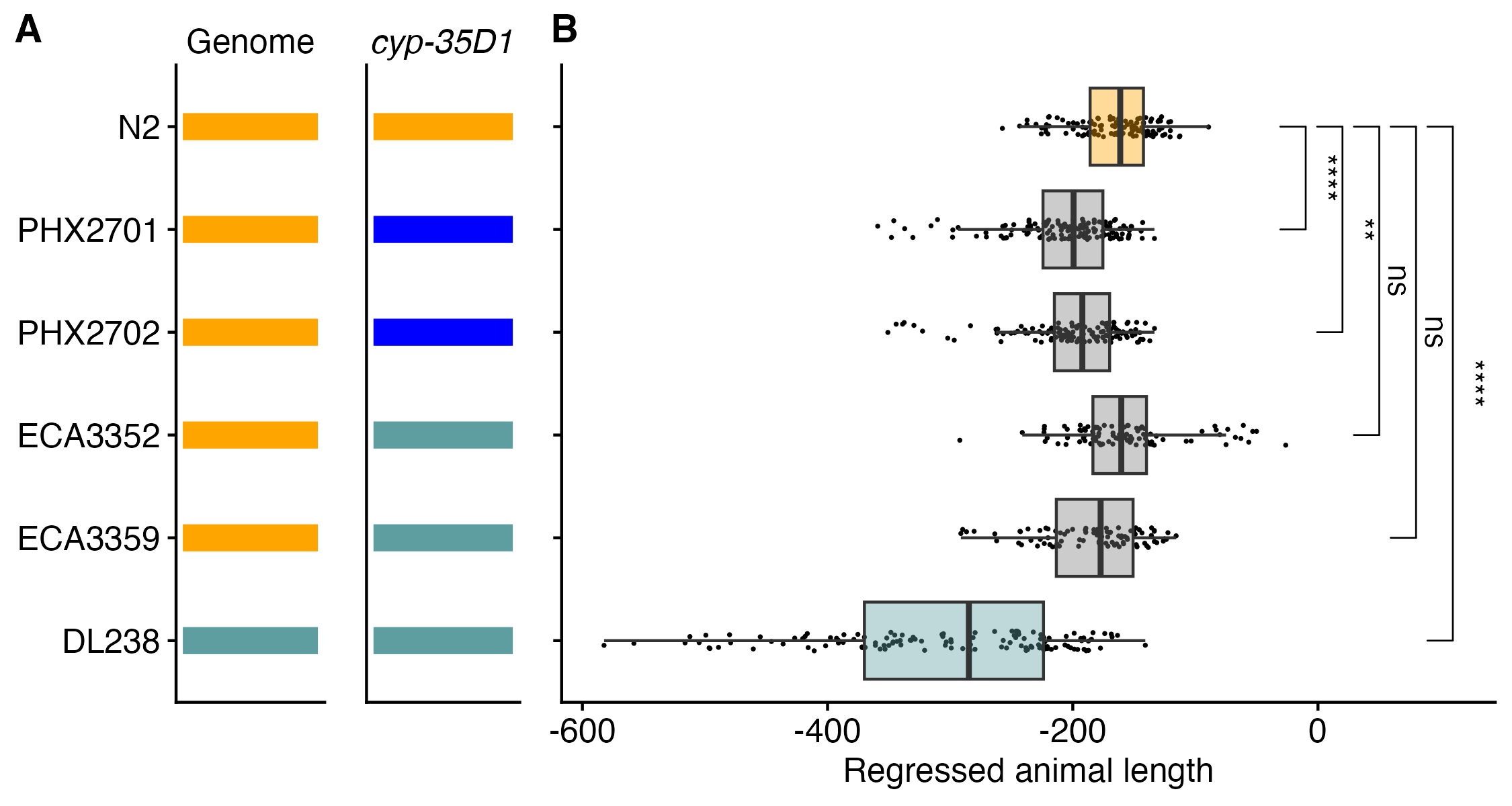

Supplement: S9 Fig — The strain name is displayed on the y-axis. Two independent edits for each allele change are shown. (A) The genomic background of the tested strain is shown as orange or cadet-blue for N2 and DL238, respectively. Alleles of CYP-35D1 are shown in the same color as the relevant parental genotype, and strains with an N2 background but with the glutamate allele are shown in blue. (B) Regressed median animal length values of response to TBZ are shown on the x-axis Each point represents a well that contains ~30 animals after 48 hours of exposure to TBZ. Data are shown as box plots with the median as a solid vertical line, the right and left of the box representing the 75th and 25th quartiles, respectively. The left and right whiskers are extended to the maximum point that is within 1.5 interquartile range from the 25th and 75th quartiles, respectively. Statistical significance in com parison to the genomic background strain is shown above each strain (p > 0.05 = ns, p <0.01 = **, p < 0.0001 = ****, Tukey HSD). (TIF) [file ppat.1012602.s028.tif]

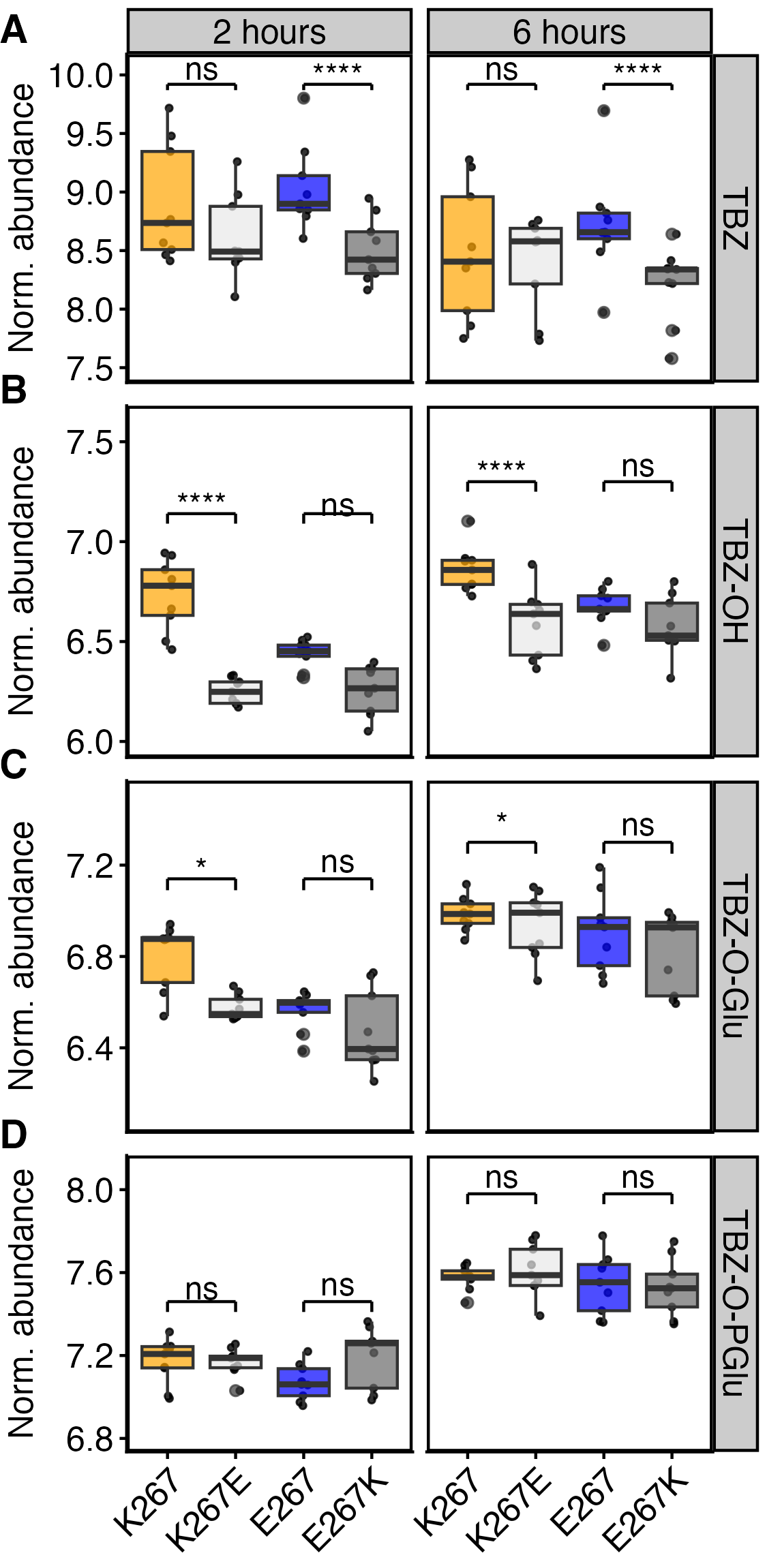

Supplement: S10 Fig — The change in the abundances of TBZ (A) and three metabolites: TBZ-OH (B), TBZ-O-glucoside (C), and TBZ-O-phosphoglucoside (D) are shown, with samples taken at two and six hours after exposure to 50 μM TBZ. CYP-35D1 alleles are shown on the x-axis with K267 (orange) and K267E (light gray) in the N2 genetic background, and E267 (blue) and E267K (dark gray) in the CB4856 genetic background. Abundances are shown as the log of abundance after normalization for the abundance of ascr#2. The line represents the mean abundance, and each point represents an individual replicate. Statistical significance between strains with the same genetic background at the same time point is shown (p > 0.05 = ns, p < 0.05 = *, p < 0.0001 = ****, Wilcoxon Rank Sum test with Bonferroni correction t-test of Independent Replicates). (TIF) [file ppat.1012602.s029.tif]

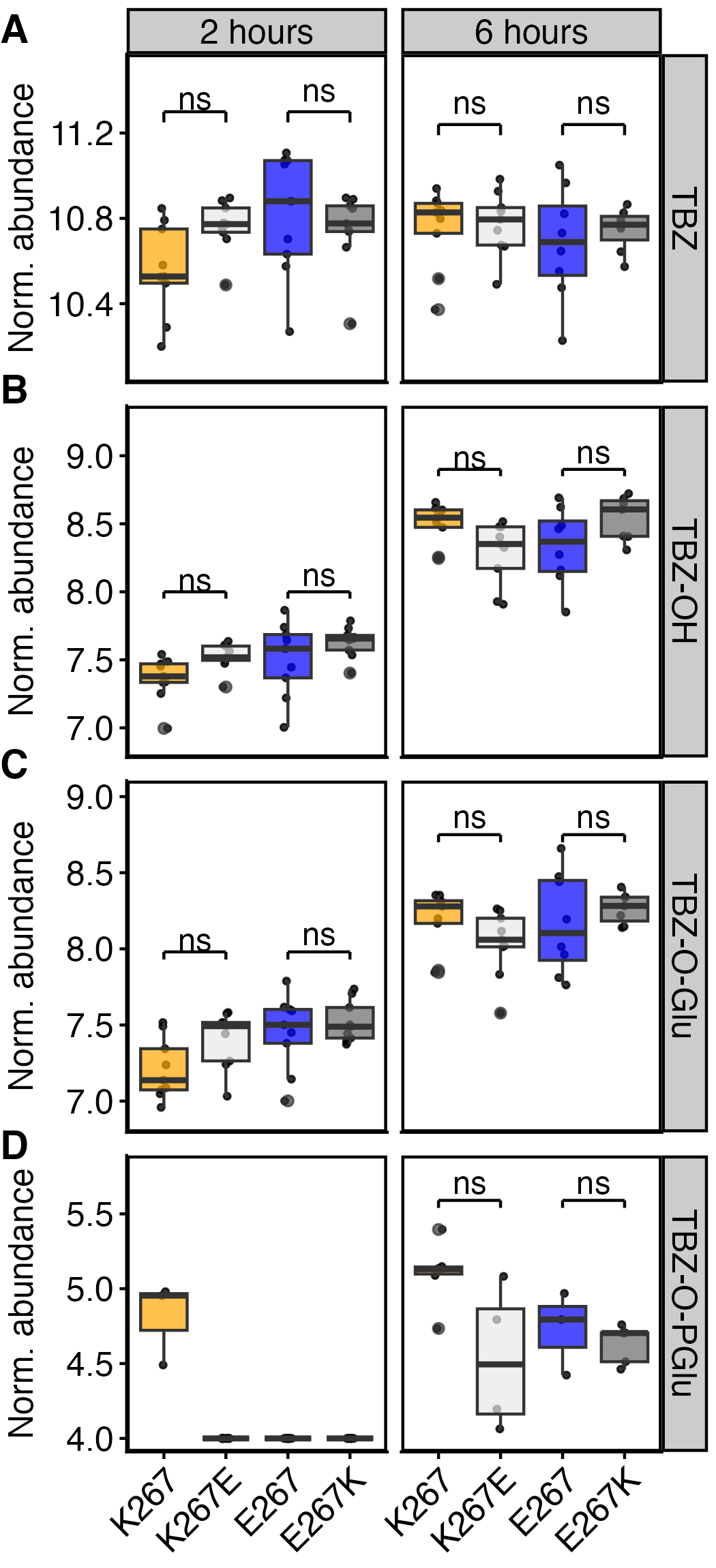

Supplement: S11 Fig — The change in the abundances of TBZ (A) and three metabolites: TBZ-OH (B), TBZ-O-glucoside (C), and TBZ-O-phosphoglucoside (D) are shown, with samples taken at two and six hours after exposure to 50 μM TBZ. CYP-35D1 alleles are shown on the x-axis with K267 (orange) and K267E (light gray) in the N2 genetic background, and E267 (blue) and E267K (dark gray) in the CB4856 genetic background. Abundances are shown as the log of abundance after normalization for the abundance of ascr#2. The line represents the mean abundance, and each point represents an individual replicate. Statistical significance between strains with the same genetic background at the same time point is shown (p > 0.05 = ns, Wilcoxon Rank Sum test with Bonferroni correction t-test of Independent Replicates). (TIF) [file ppat.1012602.s030.tif]

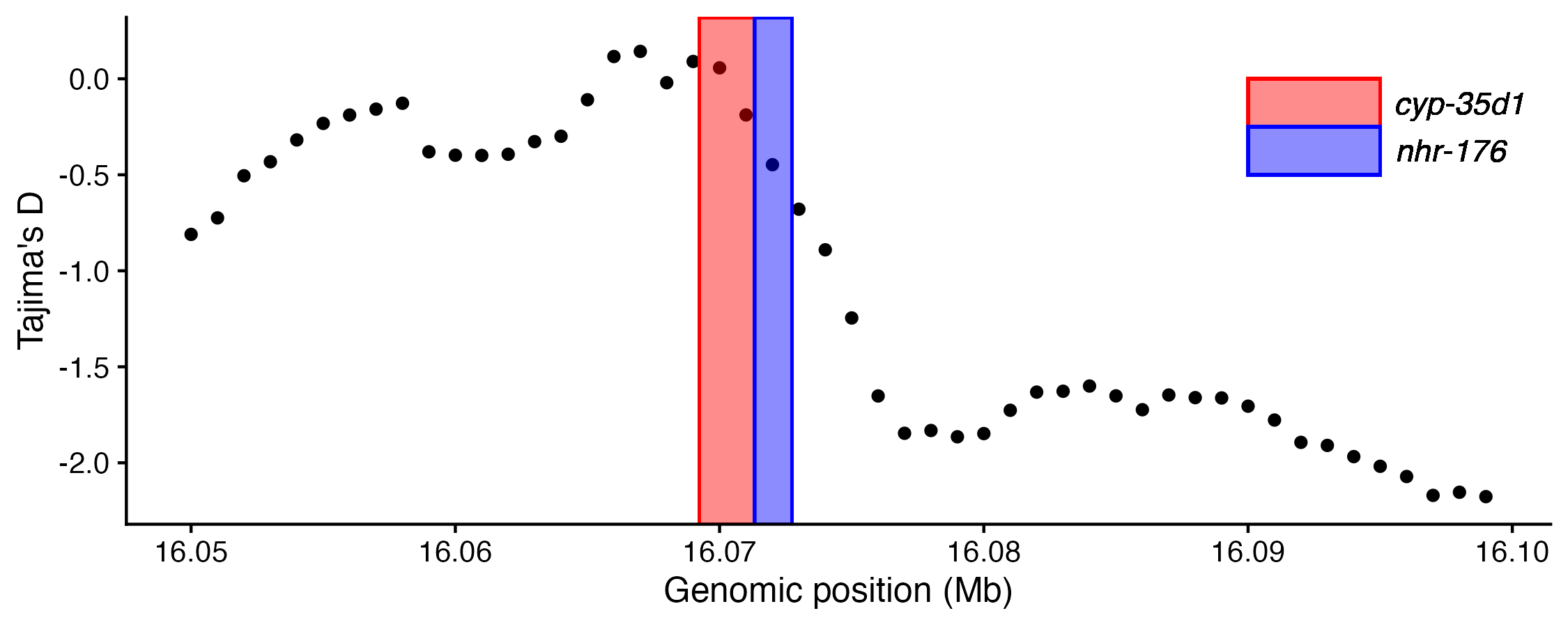

Supplement: S12 Fig — The divergence measured by Tajima’s D surrounds the cyp-35D1 and nhr-176 locus on chromosome V. The most recent CaeNDR variant release was used to calculate Tajima’s D in this region [31,58]. (TIF) [file ppat.1012602.s031.tif]
