# Supplementary material for: Naturally occurring variation in a cytochrome P450 modifies thiabendazole responses independently of beta-tubulin
Source: PLoS Pathog. 2025 Jan 14;21(1):e1012602. doi: 10.1371/journal.ppat.1012602 (PMC11771912; doi:10.1371/journal.ppat.1012602)
Supplement: Attachment — Submitted filename: 2024TBZ_Response_to_Reviewers.docx [file ppat.1012602.s032.docx]

Dear Editor,

Thank you for considering our manuscript “Naturally occurring variation in a cytochrome P450 modifies thiabendazole responses independent of beta-tubulin.” We thank the editor and reviewers for their comments. We have addressed the comments in the text (quoted below) and offer responses to the major comments below. Thank you for considering our revised submission.

**Major Comments:**

*Reviewer 1:*

**“Dosage of thiabendazole (TBZ) used is not always clearly presented. The dosages used are never compared to dosages used medically or agriculturally or encountered environmentally, a question of obvious relevance that is never mentioned. No comparison is made between the level of TBZ resistance achieved through the cytochrome 267K polymorphism and that possible through mutation of the tubulin gene ben-1. A dose-response assay would seem ideal, comparing relevant cyp-35D1 genotypes to each other and potentially to ben-1 mutants, but at the least this should be discussed and should be considered more thoroughly.”**

Thank you for this comment. We have made edits throughout so that we are clear on the dosages of TBZ used. Comparisons between *in vitro* and *in vivo* concentrations are not able to be made with any accuracy because data on drug concentration within the host in a given tissue or organ system are not well established. The same problem exists for environmental levels. Furthermore, concentrations used in laboratory *in vitro* assays rarely correspond to physiological concentrations due to differences in exposure dynamics. Lastly, we have better clarified that the level of resistance conferred by *ben-1* mutations is much higher than levels conferred by *cyp-35D1.* Additionally, we added further detail on how even the lower levels of resistance associated with *cyp-35D1* could be important in the field.

“Although the effects of variation in CYP-35D1 are relatively small compared to the impact of variation in the beta-tubulin target of BZs, changes to drug metabolism could have significant impacts on the evolution of resistance in a susceptible population. Inadequate dosing is all too common, and the increased metabolism conferred by variation in a metabolic pathway could enable parasite survival until a larger effect mutation is acquired, such as mutations in beta-tubulin.“

**At one point in the manuscript (circa line 434) the authors state that although they have used TBZ throughout this study, another related molecule fenbendazole (FBZ) is now in much greater use than TBZ. For example, the H. contortus isolates examined for cyp-35D1 homolog mutations had been treated not with TBZ but with FBZ. It would be good to show whether mutations of cyp-35D1 can affect susceptibility to FBZ in C. elegans. This also applies to albendazole (ABZ), which is only mentioned once in the manuscript to cite a reference.**

We have added additional information, highlighting that in previous published and unpublished genetic mapping experiments, using albendazole and fenbendazole, the *cyp-35D1* QTL is not detected. For this reason, we did not go further with these other two BZs. We have added a statement in the Discussion making this clearer:

“Furthermore, previous GWAS mappings using fenbendazole and albendazole did not identify the *cyp-35D1* QTL, although other novel QTL were identified in these mappings [(Zamanian et al. 2018; Hahnel et al. 2018)](https://paperpile.com/c/r0ttsh/XXrq+DsQr).”

**The authors demonstrate that under laboratory conditions, over a few generations, there might not be any deleterious or advantageous effect of the cyp-35D1 267K polymorphism in competition assays. This result could be very important, but the data indicating it seem to be limited. Laboratory culture conditions are not highly selective. Furthermore, it isn’t clear from the authors’ presentation of the data (Figure 3A) whether there is indeed no difference between the 267E and 267K alleles; the authors report no statistically significant difference, but there appears possibly to be both increased variation and a downward trend from the 267E allele. It is possible that an increased number of trials or an increase in number of generations would result in this trend achieving statistical significance – which could in turn explain why the 267K polymorphism became fixed over many generations of culturing C. elegans under these same laboratory conditions prior to the strain first being frozen. The authors also do not report whether there is any effect of cyp-35D1 deletion alleles, either in competition assays or by measuring brood size and time to maturation. Similarly the authors do not discuss whether there are any reported effects on health or fecundity of TBZ resistance mutations in ben-1.**

We agree with the reviewer's point that more generations of competition could have different results. However, the downward, not statistically significant trend, is present in both strains, leading us to believe the observed decrease is likely not biologically significant. We did not test the effects of deletion alleles in the competition assay, because the variant tested does not appear to cause loss-of-function, thereby making a deletion not representative of what is encountered in nature. Previous publications from the Andersen lab have documented a lack of effects of *ben-1* mutations on fitness, and we have added a brief clarification comparing the two genes in terms of competitive fitness.

“Similar to previous studies of effects of *ben-1* mutations on competitive fitness, we found that fitness of strains with the *cyp-35D1* resistance allele are not unfit in control conditions, indicating that once the allele emerges in a population, it will be maintained in the absence of selection.”

”**The authors report looking for cytochrome P450 changes corresponding to the 267K polymorphism in the genomes of selected Caenorhabditis nematodes and by sequencing samples from 128 samples of FBZ-exposed populations of the parasitic nematode H. contortus. In none of these do they report finding a 267K polymorphism, including none arising in the FBZ-exposed H. contortus (in which context it seems again relevant that the authors do not report having tested whether C. elegans with the 267K genotype are resistant to FBZ). The authors should look more systematically at nematode genomes, and should more thoroughly describe the cytochrome P450 phylogeny in nematodes. An admittedly sloppy survey of Caenorhabditis finds that the first BLAST hit when querying with C. elegans CYP-35D1 often – perhaps half of the time – finds an asparagine at the position corresponding to amino acid 267 of CYP-35D1.**

**One of the four H. contortus homologs of CYP-35D1 that the authors describe also has this 267N sequence, which the authors note but do not discuss. It’s possible these homologous proteins (both the Caenorhabditis homologs with 267N and the H. contortus protein with 267N) are orthologous to cytochromes of C. elegans other than CYP-35D1 (there is at least one C. elegans cytochrome with an asparagine at this position),**

**but the authors should discuss this. Moreover, 267K sequence can be found in at least two other nematode species (the pine tree nematode Bursaphelenchus okinawaensis and the mammalian parasite Strongyloides stercoralis), and still other amino acids can be found in this position in highly ranked BLAST hits from other available nematode genomes (Q in Deladenus siricidicola, Nippostrongylus brasiliensis, Poikilolaimus oxycercus, and Steinernema carpocapsae; also G in Steinernema carpocapsae; and P in Panagrellus redivivus).**

**It is striking that the authors experimentally investigated the 267D polymorphism found in one isolate of C. elegans (a highly conservative change from 267E that can be found in many homologs of CYP-35D1 throughout nematodes) but do not discuss any other variants, not even the asparagine variant they report having found in H. contortus.**

We agree with the reviewer that additional variants are present across a number of species. However, predicted orthology outside of clade V nematodes is highly diverse. Therefore, we focused on variants identified in *C. elegans* that we could mechanistically test the effects on metabolism in *C. elegans*. We have better clarified the alleles observed in the species shown in Fig 7, as well as briefly highlighted the presence of additional variants in other species in the Discussion. We propose broader exploration of the cytochrome P450 family as a future direction.

“Position 267 of CYP-35D1 and related orthologs do not have the TBZ-resistant lysine allele in any orthologous gene across the *Caenorhabditis* genus (Fig 7B). Glutamate and aspartate are the most common alleles identified in the *Caenorhabditis* species, except for serine in *C. bovis* and asparagine in *C. becei*. The four most closely related *H. contortus* orthologs also did not have a lysine at position 267 but did have either aspartate or glutamate (Fig 7B) [(Stephen R. Doyle et al. 2020)](https://paperpile.com/c/r0ttsh/BnkdS).”

“However, it is important to note that other nematode species like *Strongyloides stercoralis* and *Bursaphelenchus okinawaensis* also have lysine at position 267 in predicted *cyp-35D1* orthologs. Additional variants at this position are also found in predicted *cyp-35D1* orthologs in other species, but the high levels of conservation of cytochrome P450s make functional determination of differences difficult. Therefore, we focused on *C. elegans* variants where we could mechanistically test the effects of each allele and search in the available FBZ-resistant *H. contortus* populations. Future efforts can explore the role of the cytochrome P450 family in BZ responses.”

**nhr-176 is mentioned extremely briefly in the manuscript, is squarely in the region implicated by genetic mapping, and according to the literature described by the authors is directly relevant to TBZ resistance, but almost as soon as it is mentioned nhr-176 is discarded from the manuscript, a choice that is never fully explained. This is particularly striking on line 110 (“We further narrowed this region to two candidate genes including the cytochrome P450 [gene] cyp-35d1”). The other gene in that region is nhr-176, which is not mentioned. At no point do the authors clearly state that nhr-176 is immediately adjacent to cyp-35D1 and even appears at a glance to be co-transcribed with cyp-35D1 in a single operon.**

The GWAS identified a broad QTL encompassing *cyp-35D1* and *nhr-176*, both of which had previously been implicated in TBZ response. Expression of both genes in N2 and CB4856 are similar indicating that expression differences likely do not underlie response. Both genes harbor predicted high-impact variants with a minor allele frequency (MAF) greater than 5%. Linkage mapping identified an overlapping QTL, with the only high-impact variant being the K267E substitution in CYP-35D1. Given that linkage mapping pinpointed a correlation only with *cyp-35D1*, we hypothesized that this cytochrome P450 underlies the observed differences in TBZ response. This hypothesis led us to focus on narrowing down the gene to a specific variant and validating its role using NILs and genome editing. We have clarified our reasoning throughout the manuscript and emphasized that examining additional *nhr-176* alleles represents an important future direction.

“No *nhr-176* variants were identified in the parental strains used for linkage mapping, but we identified a lysine-to-glutamate substitution at position 267 (K267E) in CYP-35D1 with lysine conferring a greater level of resistance to TBZ.“

“Multiple variants with minor allele frequencies greater than 5% were found in both *cyp-35D1* and *nhr-176* in strains from the GWAS population. However, linkage mapping identified the same QTL in populations where only a lysine-to-glutamate substitution at position 267 in CYP-35D1 was found. Because linkage mapping pinpointed a correlation only with *cyp-35D1*, we hypothesized that this cytochrome P450 underlies the observed differences in TBZ response.”

“Also, although we did not identify variation in the nuclear hormone receptor NHR-176 in the CB4856 strain, further exploration of the second gene in the QTL would be interesting. Variants in *nhr-176* found in other strains are present and could alter the TBZ response. Additionally, differences in expression of *cyp-35D1* and *nhr-176* are not found between CB4856 and N2. However, expression differences in other strains could also play a role in altering the TBZ response.”

**Speaking of the possibility that nhr-176 and cyp-35D1 might be in an operon: the authors report generating deletion mutants of each gene, but do not report whether either deletion allele impairs the other gene, either by looking for the expression of the mRNA or by using complementation to test the function of the other gene. The authors mutate both genes separately in cis, without first demonstrating whether they had already functionally done so by disrupting the potential operon.**

From operon data collected on WormBase for *C. elegans*, *cyp-35D1* and *nhr-176* are not listed as part of a known operon. Deletion of one gene or both results in equivalent phenotypes for TBZ response, indicating that loss of either one of the two genes is sufficient to confer increased susceptibility. However, we can only make the conclusion for TBZ response, because both genes are likely part of additional pathways with likely pleiotropic effects.

“Although the genes are side by side in the genome, they are not part of an operon and were examined separately.”

**The authors do not report testing whether TBZ resistance caused by the 267K polymorphism of cyp-35D1 is dominant or recessive, at any dosage of TBZ, nor do they state what is known about dominant or recessive action of ben-1 mutations. This seems of obvious importance, especially for parasitic nematode species that might homozygose newly emerging mutations less quickly than does the selfing hermaphrodite C. elegans.**

We thank the reviewer for this comment and recognize that dominance is an important facet in the spread of resistance. Previous work from the Andersen lab highlighted that mutations associated with resistance in *ben-1* are recessive, suggesting that resistance might spread slowly. However, it is not known if these results are consistent in parasitic species due to potential differences in how resistance is conferred by mutations given different complements of beta-tubulin genes. The experiments to test dominance are difficult, necessitating hand selection of heterozygotes and integration into highly scaled high-throughput assays. For this reason, we did not perform these experiments. However, it would be interesting to test in the future if we can identify further relevance in field populations of parasites. We added text to the Discussion to address this point.

**“**Similar to previous studies of the effects of *ben-1* mutations on competitive fitness [(Dilks et al. 2020)](https://paperpile.com/c/r0ttsh/4rhJ), we found that the fitness of strains with the resistance allele are not significantly selected against in control conditions, suggesting that once the allele emerges in a population, it will be maintained in the absence of selection. We do not know if *cyp-35D1* resistance is dominant, and future experiments can address this point. Resistance phenotypes conferred by *ben-1* were previously shown to be recessive [(Dilks et al. 2021)](https://paperpile.com/c/MS1X1I/tCrp).”

**The authors report different levels of activity when different versions of CYP-35D1 are expressed in yeast from identical plasmids. They don’t report doing anything to confirm identical levels of protein expression or stability in their transgenic yeast. I believe the plasmids they used should avoid issues of plasmid copy number, but even so the reader should be reassured that these potential caveats have been addressed experimentally or at least that they have been considered and discarded according to the standard of the field.**

Any differences between the transgenic yeast lines are greater than the differences between the plasmids as the statistical analyses show. These results suggest that the differences pointed out by the reviewer are experimental noise. We have added the following point to the Figure description to emphasize these results.

“**Figure 5. Activity of wild-type and mutant CYP-35D1 expressed in yeast.** The metabolic activity of the lysine and glutamate versions of CYP-35D1 when expressed in yeast and exposed to 100 µM TBZ for six hours is shown. The activity of each enzyme is shown relative to the wild-type activity. Each point represents an individual replicate. Data are shown as box plots, with the median as a solid horizontal line and the top and bottom of the box representing the 75th and 25th quartiles, respectively. The top and bottom whiskers are extended to the maximum point that is within 1.5 interquartile range from the 75th and 25th quartiles, respectively. The top and bottom vertical whiskers extend to the maximum point within 1.5 interquartile range from the 75th and 25th quartiles, respectively. Significant differences between the lysine and glutamate allele are shown as asterisks (*p* > 0.05 = ns, *p* < 0.05 = *, Wilcoxon Rank Sum test with Bonferroni correction). Differences observed between replicates of the same transgenic yeast line exceed any observed differences between different lines.”

**Minor Issues:**

*Reviewer 1:*

**Reviewer #1: 9) According to WormBase the C. elegans gene name is “cyp-35D1”, not (as it consistently appears in the manuscript) “cyp-35d1”. I believe the H. contortus gene homologous to C. elegans ben-1 may be “Hco-tbb-iso-1” (it appears in the literature in that way at least once), but it surely cannot be “Hco-isotype-1”, as it appears in this manuscript at least twice.**

We have corrected to *cyp-35D1* and *Hco-tbb–iso-1* throughout the manuscript.

**10) Colors are used in histograms in the Figures that are frequently not mentioned in the legends and are not obviously consistent from figure to figure.**

We have tried to better clarify colors throughout the figure legends. We also modified colors to be consistent throughout.

**11) Line 83: It is striking that the introduction considers parasitic nematodes and their relevance to human and veterinary health, but does not mention plant-parasitic nematodes and their control in agriculture. This might easily be relevant to the emergence of TBZ-resistant phenotypes in free-living soil nematodes such as C. elegans, and also to the presence of a polymorphism equivalent to cyp-35D1 267K in the plant-feeding nematode Bursaphelenchus okinawaensis. The authors similarly do not mention the use of TBZ and related drugs against parasitic invertebrates other than nematodes.**

Although an interesting point, we feel that this point is beyond the scope of the present manuscript focused on using *C. elegans* as a model for studying anthelmintic resistance in parasitic nematodes of vertebrate hosts.

Notably, *C. elegans* is NOT a soil nematode as is often mistakenly stated in the literature.

**12) Line 142: The text says “Highly TBZ-resistant strains had predicted loss-of-function variation in ben-1 (Fig 1A)”. Every strain in Figure 1A marked with a red triangle (defined as “with variation in ben-1”) demonstrates increased sensitivity to ben-1, not increased resistance. This is confusing.**

We thank the reviewer for the comment. The label on the Y-axis is an oversight and has been corrected to “TBZ Resistance”.

**13) Line 148: List the six beta tubulin genes, here or in the Methods.**

We have added the requested information.

“Variation in *ben-1* is known to play a large role in the BZ response, but our goal was to identify novel resistance mechanisms independent of the beta-tubulin genes (*tbb-1*, *tbb-2*, *mec-7*, *tbb-4*, *ben-1*, *tbb-6*).”

**14) Line 152: The text here attributes to reference 19 a distinction between the QTLs on LGII and on LGV in their interactions with the ben-1 locus. This distinction is not obviously made in reference 19, which states that controlling for variation in ben-1 “resulted in the disappearance of the two QTL on chromosome II and the QTL on chromosome V”. Please clarify.**

We have deleted a sentence and simplified to the following for clarity:

**“**The lack of the chromosome II QTL in the *ben-1-*regressed mapping suggests that this QTL is associated with variation in *ben-1*, which has also been previously documented in genetic mappings using albendazole [[19]](https://paperpile.com/c/Niyn5W/YJRN2),...”

**15) Line 183 (Figure 1): The label in the figure “Neither” could be clearer. The legend should state that 214 strains are shown.**

We have removed the “Neither” label because we are only showing where N2 and CB4856 are on the distribution. We have also clarified in the figure legend that 214 strains are shown.

“(A) Distribution of normalized relative resistance in 62.5 µM TBZ is shown in order from most susceptible to most resistant for 214 wild *C. elegans* strains.”

**16) Line 272: The manuscript states that introduction of the 267E polymorphism to the N2 strain did not appear to reduce TBZ metabolism, even though the corresponding and opposite change in CB4856 increased metabolism. This is briefly stated and is never discussed.**

Comparisons of strains in different genetic backgrounds (*i.e*., N2 and CB4856) cannot be accurately made because the metabolic flux is different between each strain and is affected by much more than just the region of interest. Instead, we focus solely on the differences conferred by the edited allele compared to the wild-type of the parental background.

**17) Line 277: “yeast” should be “Saccharomyces cerevisiae”. Also, a glance at the literature indicates that expression of metazoan cytochromes in yeast to test their metabolic function is a standard method, but this is not made obvious in the manuscript, nor is it mentioned why it’s seen as an appropriate system – and unless I have overlooked it none of this literature seems to have been cited in the Results, the Discussion, or the Methods.**

We have added the following statement.

“To determine if the observed metabolic effects could be recapitulated in another model system, we generated strains of *Saccharomyces cerevisiae* (yeast), a system previously used to measure CYP-35D1 mediated metabolism of cyprocides [(Knox et al. 2024)](https://paperpile.com/c/MS1X1I/sKzy),...”

**18) Line 285 (Figure 4): Four asterisks is not defined in the legend. Strain backgrounds could be more clearly labeled, rather than being inferred from starting genotype.**

We have added the label for four asterisks. We have also provided more detail for the strains in the legend: “K267 (orange) and K267E (light gray) in the N2 genetic background, and E267 (blue) and E267K (dark gray) in the CB4856 genetic background.”

**19) Line 318: Should “area” be plural? I am confused by this statement.**

We have revised for better clarification.

“Using data available from the *Caenorhabditis* Natural Diversity Resource [(Crombie et al. 2024)](https://paperpile.com/c/r0ttsh/VoWCf), we found that all three alleles are broadly distributed, found both in the same geographical areas, as well as across multiple continents [[28]](https://paperpile.com/c/Niyn5W/8Yz2) (Fig 6A).”

**20) Line 320: This dendrogram is offered to the reader, but is never explained to them. Does it indicate a single, relatively recent origin of the 267K polymorphism, that has been spread rapidly? How much more information or at least argumentation can be extracted from this dendrogram?**

The dendrogram in part B of the figure represents the relatedness of the strains shown in part A. The broader geographical distribution of the glutamate allele suggests that the lysine allele is a more recent evolutionary development. Furthermore, the large number of strains with the lysine allele, compared to those with the glutamate and aspartate alleles, supports the hypothesis that the lysine allele confers a fitness advantage and is likely to persist once selected for in a population. We have revised the manuscript to improve clarity in its interpretation.

“The broader geographical distribution of the glutamate allele, along with its high frequency across nematode orthologs of CYP-35D1, suggests that glutamate represents the ancestral allele, and the lysine allele is likely a more recent adaptation. The significant number of strains carrying lysine at position 267 indicates that this allele is being selected and, once selected, will likely persist in populations.”

**21) Lines 322-325: This is an argument to be made in the Discussion, not the Results. Also, given that the 267K polymorphism appears to be at worst neutral, the argument should be made at greater length, potentially citing relevant work.**

We agree and have moved this statement into the larger relevant context of the Discussion.

**22) Line 328 (Figure 6): In designing figures the authors should consider the incidence of color-blindness. Also, as mentioned previously, extremely little effort is made to explain the figure to the reader or to discuss its significance. This is frequent throughout the manuscript.**

We have modified the colors for the alleles to be consistent with the colors used for the parental genotypes elsewhere in the manuscript.

. **23) Line 398: The authors propose that the TBZ-resistant cyp-35D1 polymorphism might have arisen in the strain N2 and become common in isolates more recently found around the world because of the presence in the environment of human-used TBZ and related drugs, and also naturally occurring related molecules. Please clarify when use of TBZ or related molecules began, and whether this could explain why a C. elegans isolate obtained in Bristol more than five decades ago and subsequently frozen has this TBZ resistance polymorphism.**

We do not specifically say that the allele arose in the N2 strain and spread. Rather, the available data indicate a more recent evolution of the lysine allele across multiple populations globally. As to the question of when commercial BZs were first used, TBZ was first approved in 1969, after the initial isolation of N2 in 1963, indicating that selection for the lysine allele occurred before the first use of commercial BZ anthelmintics. We have clarified the order of events in the manuscript.

“Interestingly, the evolution of the more active CYP-35D1 enzyme appears to predate commercial use of BZs, because the lysine allele was identified in the N2 strain, which was first isolated in 1951, 18 years prior to the release of TBZ. Natural BZs in the environment have likely led to selection for the resistant allele in CYP-35D1, corresponding with the significantly higher relative fitness of the lysine allele compared to the glutamate allele in treatment conditions.”

**24) Line 423: I assume “predicted high impact” means a nonconservative coding change?**

We have clarified that this description is from variant annotation software and denotes an amino acid change or nonsense codon addition.

“Data available in the *Caenorhabditis* Natural Diversity Resource show multiple CYP-35D1 variants that alter amino acid residues in both…”

**25) Line 424: “although we can draw no conclusions about the resistance status”. This seems like it could be overcome, if this were desired.**

Although true that gene editing can be done in other selfing *Caenorhabditis* species, BZ resistance in the two additional selfing species has not been characterized as in *C. elegans*. Characterization of how *cyp-35D1* variants alter responses in *C. briggsae* and *C. tropicalis* are part of a bigger study to determine if the mechanisms of resistance are conserved across the three selfing *Caenorhabditis* species.

**26) Line 434: Expand on the statement about the discontinued use of TBZ. Why was it stopped? When was it stopped? Is the cyp-35D1 267K polymorphism specific to TBZ, versus other related drugs?**

We have revised this section to the following.

“However, it is important to note that TBZ has not been used in the field for many decades, caused in part by the creation of more effective BZ compounds. The tested *H. contortus* populations have been exposed to heavy selection with fenbendazole (FBZ). Furthermore, previous GWAS mappings using FBZ and ABZ did not identify the *cyp-35D1* QTL, although other novel QTL were identified in these mappings (Zamanian et al. 2018; Hahnel et al. 2018).”

**27) Line 465: This section is unclear. Were 219 new RIAILs generated for use in this study, according to the methods described in reference 22, or is this study referring to a subset of the 359 RIAILs generated in that referenced work? Were the sequence reads deposited in the Short Read Archive?**

A subset of previously generated RIAILs was used. All data associated with these RIAILs are available as part of the original cited manuscript [(Andersen et al. 2015)](https://paperpile.com/c/r0ttsh/EPS3).

**28) Line 471: Despite the statement that all strains are listed in Supplementary File 1 and are available from the CaeNDR, Supplementary File 1 does not list 219 RIAILs, does not seem to obviously list any RIAILs. Were the RIAILs generated for this study, or not? If so, were they all preserved? Were any preserved?**

We have added the RIAILs used to S File 1, and strains are preserved and available through CaeNDR.

**29) Line 471: Supplementary File 1 does not appear to list the cyp-35D1 nhr-176 double mutants used in Figures S5 and S6.**

All strains, including RIALs, are now listed in S File 1.

**30) Line 473: This paragraph does not mention the generation of the double deletion mutants used in Figures S5 and S6.**

We have added this information in the following paragraph: “Additional strains with single gene deletions of either *nhr-176* or *cyp-35D1*, deletions of both genes, as well as strains with a K267D substitution, were generated using CRISPR-Cas9 genome editing as previously described [16,19] Double deletions were generated by deleting *cyp-35D1* in the ECA2924 *nhr-176(ean226)* deletion background.”

**31) Line 473: Mention here in the Methods that oligonucleotide sequences are in Supplementary File 1.**

We have added the sentence.

“All oligonucleotides used for CRISPR-Cas9 editing and confirmation are found in S File 18.“

**32) Line 473: Oligos listed in Supplementary File 1 include the “First crispr guide for cyp-35D1 deletion” but not any second guide, nor any oligonucleotide or construct used to guide repair, nor do they list any reagents for the nhr-176 deletions.**

We have added this information to S File 18.

**33) Line 477: The sequence changes in the deletion alleles are not described (and as mentioned above the sequences of the reagents used to generate them are not provided).**

As noted in the last comment, we have added the information to the S File 1 as part of the strain list. In addition, we have noted the amino acid changes made by the edits.

**34) Line 485: Give or cite a detailed protocol for “bleach synchronized”.**

We have cited a link to the Andersen lab website for the protocol.

“(Protocol available at <https://andersenlab.org/Research/Protocols/>)”

**35) Lines 488 and 490: “L1” and “L4” are not defined.**

We have defined the terms on the first use of L1 and L4.

“...L1 larvae (first larval stage) were fed lyophilized bacterial lysate (*E. coli* HB101 strain) at 5 mg/mL in K medium. Nematodes were grown for 48 hours at 20˚C with constant shaking at 180 rpm. Three L4 larvae (fourth larval stage) were…”

**36) Line 628: Name, cite, and/or acknowledge the codon optimization tool used. Provide codon-optimized sequences in the Supplementary Files or upon request. Provide more information about site-directed mutagenesis: cite a reference or name a kit, at least. Note that any oligonucleotides used in this section are not included in Supplementary File 1.**

We have listed the kit used and have added the oligos used to S File 18.

“Q5 Site Mutagenesis Kit, New England Biolabs, Ipswich, MA, USA”

**37) Line 693: Provide accession numbers or other identifying information for the sequences used.**

We have added this information as part of S File 19.

“All relevant WormBase accessions for *cyp-35D1* (and orthologs) sequences used can be found in S File 19.”

**38) Line 746: WormBase should be cited according to the FAQ on their website.**

We have indicated that we used WormBase for these sequencing data and used the relevant citation in the main manuscript. “Orthologs of *cyp-35D1* were identified in the three parental *C. elegans* strains used in this study (N2, CB4856, and DL238) and from representative strains from multiple supergroups within the *Caenorhabditis* genus using BLAST and the WormBase database [(Stevens et al. 2020; Stevens et al. 2019; Sternberg et al. 2024)](https://paperpile.com/c/r0ttsh/h7UR+6SMT+L5SP).”

**39) Line 983 (Figure S2): Legend should do more to explain the figure to the reader.**

We have revised to specify what is meant by the REF and ALT alleles, as well as what information if being shown for the y-axis in terms of phenotype. We have also clarified that each point represents an individual strains normalized response from multiple replicates.

**“**(A) Regressed animal length (mean.TOF) values in response to TBZ treatment in a genome-wide association study are shown on the y-axis. The x-axis denotes if a strain has the lysine (REF) or glutamate (ALT) alleles at the peak marker. Each point represents a strain’s normalized response from multiple replicates. (B) For the QTL discovered in the linkage mapping experiment, the regressed animal length (mean.TOF) is shown on the y-axis, and the allele of the tested recombinant strain is shown on the x-axis. Data are shown as box plots with the median as a solid horizontal line, the top and bottom of the box representing the 75th and 25th quartiles, respectively.”

**40) Line 1009 (Figure S4): Black angle-bracket shapes (gene models maybe?) are not mentioned in the legend, and nhr-176 is not marked in the figure.**

We have clarified that “ black arrows indicate the direction of transcription”.

**41) Line 1016 (Figure S5): Top and bottom rows below the figure should be labeled in the figure, not merely described in the legend. “WT” should be defined in the legend. Allele numbers should be stated, not merely “del”, considering that the whole point appears to be that two different deletion alleles were used. The 2nd, 3rd, 7th, and 8th rows all have equivalent genotypes, but the reason for this, and for the comparisons being made, is not clear. Yellow and blue colors in histograms not explained.**

We have made the suggested modifications to the supplemental figures. We highlight in the existing legend that the data shown are of each independent replicate of the two. We have added the clarification of “WT” and the colors.

“The strain *cyp-35D1* genotype (top) and *nhr-176* genotype (bottom) are shown on the x-axis, with WT representing the wild type for each gene. N2 (orange) and CB4856 (blue) are shown for comparisons with genetic backgrounds. “

**42) Line 1044 (Figure S6): Comments to Figure S5 apply.**

We have made the edits to S Fig 6 as done for comment 41 (S Fig 5).

**43) Line 1061 (Figure S7): Single asterisk not defined in legend. Comparisons are not “above each strain”. Strain names should be labeled as strain names in the figure. Legend should clarify whether these are introgression strains or CRISPR strains.**

We have modified the legend to include the single asterisk and that the comparisons are to the right. We have also clarified that the data are from CRISPR-Cas9-edited strains in the figure title.

**44) Line 1095 (Figure S9): Dark blue not defined in legend with other colors. Typo in figure heading. Figure legend refers to a part C not in evidence. Parts A and B are not separate from each other.**

We have added this clarification: **“**Alleles of CYP-35D1 are shown in the same color as the relevant parental genotype, and strains with an N2 background but with the glutamate allele are shown in blue.”

**45) Line 1108 (Figure S10): Legend defines a two-asterisk condition not present in the manuscript.**

We removed this label.

**46) Line 1132 (Figure S12): I do not think this figure is mentioned in the manuscript.**

We added a citation to this figure and included the details in the relevant section of the manuscript.

“We investigated the haplotypes at the *cyp-35D1* locus and generated a tree for a region containing 25 kb to either side of *cyp-35D1* (Fig 6B). The tree could be divided into three distinct clades, with the lysine allele in two of the clades. Tajima’s D was calculated for the same region used to generate the tree, and found no signature of selection at the locus (S12 Fig)”

*Reviewer 2:* **Lines 340-342: The authors write, "The relative distance between the elegans and japonica groups suggests that the elegans group cyp-35d1 has recently evolved and is not closely related to cyp genes in other Caenorhabditis species." I think this is probably a mistaken interpretation that arises from a poorly-publicized defect of the C. japonica genome that is still the one official C. japonica genome in ParaSite (**[**https://parasite.wormbase.org**](https://parasite.wormbase.org/)**), for reasons explained immediately below.**

**Figure 7: The authors intelligently picked both C. japonica and C. panamensis as representatives of the japonica supergroup (as described in their Methods). Their phylogeny shows that C. panamensis has a cyp-35d1 ortholog that falls phylogenetically within the C. bovis outgroup ortholog. However, their phylogeny has one putative C. japonica ortholog that falls completely outside the otherwise monophyletic Caenorhabditis clade in their Figure 7A phylogeny.**

**I think the reason they got this strange result is that the C. japonica genome, unlike almost every other Caenorhabditis genome assembly that was produced from 2010 onward, is incomplete (despite the fact that it was assembled early and expensively by the genome center at Washington University). This fact can be easily observed by going to the species genome page at ParaSite (**[**https://parasite.wormbase.org/species.html**](https://parasite.wormbase.org/species.html)**) and looking at BUSCO scores for the 22 Caenorhabditis species' genomes on that page. There are 18 Caenorhabditis species' genome sequences that have genome BUSCO completeness scores of 97.0% to 98.6%. In contrast, the C. japonica genome assembly has a genome BUSCO completeness score of 92.4% (whereas the equivalent score for C. panamensis is 97.8%). I therefore suspect that in real life, C. japonica has a much closer ortholog of cyp-35d1 that on a phylogenetic tree would cluster very closely with the ortholog from C. panamensis. There does exist a (so far unpublished) superior C. japonica genome assembly by Lewis et al. (**[**https://www.ncbi.nlm.nih.gov/datasets/genome/GCA_963572235.1**](https://www.ncbi.nlm.nih.gov/datasets/genome/GCA_963572235.1)**) that the authors might search for a better cyp-35d1 ortholog (though they would need to manually annotate such a gene; the new and improved C. japonica genome is so far unannotated with protein-coding genes). Alternatively, the authors might replace C. japonica with some other japonica supergroup species with a better-assembled and annotated genome, and recompute their phylogeny and multiple sequence alignment in Figure 7 using a japonica-supergroup replacement for C. japonica. However, I do think that the authors should not continue taking their results with C. japonica at face value, and making statements about the evolution of this gene based on what may be only an apparently high divergence of this gene in C. japonica.**

Thank you for this information. We have replaced *C. japonica* with *C. becei*, which has the second highest BUSCO score for a species in the japonica group after *C. panamensis*. As the reviewer surmised, the issue with a member of the japonica group being more distantly related to *C. elegans* CYP-35D1 disappeared with *C. becei* clustering with *C. panamensis*. We have updated the figure, as well as modified the relevant section in the results.

“For *C. tropicalis*, another member of the *elegans* group, the closest ortholog *Csp11.Scaffold629.g12881* is more closely related to orthologs from the *japonica* group species *C. becei* and *C. panamensis*.” **POSSIBLY INTERESTING IMPLICATIONS

1. The authors show a tight association between two adjacent genes in C. elegans, cyp-35d1 and nhr-176. Examining WormBase (**[**https://wormbase.org**](https://wormbase.org/)**) for their expression data shows that both of these genes are strongly expressed in the intestine. Surai and Hobert ([2021], PubMed 34348120) showed that many nhr genes in C. elegans appear to be expressed in neurons and may act to amplify sensory responses by binding to small molecules and then upregulating seven-transmembrane receptors for those small molecules. The authors' work on nhr-176 shows an interesting counterexample in which an NHR protein is not used for sensory responses in neurons but for detoxification responses in the intestine. This may be a more general phenomenon in nematodes.**

We have added this point in the Introduction where we briefly describe the metabolic pathway.

“Interestingly, nuclear hormone receptors are often associated with neurons and sensory responses [(Sural and Hobert 2021)](https://paperpile.com/c/r0ttsh/xmjJ). However, NHR-176 is associated with the nematode intestine where TBZ binds to the receptor and then induces the expression of *cyp-35D1* [(Jones, Flemming, and Urwin 2015; Jones et al. 2013)](https://paperpile.com/c/r0ttsh/fEpK0+EQyb8).”

**2. The authors scanned many genomes of fenbendazole-resistant Haemonchus contortus isolates for lysine alleles in four H. contortus orthologs of cyp-35d1. I can confirm that orthology analysis identifies precisely these four genes, and that one of these four genes (HCON_00022670) has strongly intestine-biased RNA-seq expression (as computed from published data by Laing et al. [2013], PubMed 23985316). However, in addition to these four strict orthologs, H. contortus' genome also encodes 21 other cyp genes (defined by presence of the Pfam motif p450 [PF00067.25]), of which four show intestinal-biased gene expression. I find that three of them have more strongly intestinally-biased expression than HCON_00022670.

Gene FC FDR
HCON_00141025 8.10319755 3.11e-23
HCON_00084620 3.549094835 1.22e-12
HCON_00143950 3.39756099 1.21e-19
HCON_00022670 2.974729846 6.90e-13 # cyp-35d1 ortholog
HCON_00024010 2.678863 2.98e-14

FC == Hco_fem_gut vs. Hco_fem_worm log2FC
FDR == Hco_fem_gut vs. Hco_fem_worm FDR

It is outside the scope of this paper, but in future work, the authors may wish to scan these other H. contortus cyp genes with strongly intestinal expression for lysine alleles in fenbendazole-resistant isolates, since the selective presssure of albendazole resistance may lead to mutations in intestinal nhr genes that are not strictly orthologous to cyp-35d1. In their Discussion, the authors may wish to note the possibility that resistance alleles may arise in H. contortus cyp genes that are not strict cyp-35d1 orthologs but do have strong intestinally expression and thus are possible detoxification enzymes.**

We thank the reviewer for these insights, and we do highlight the need to look at other sites in more orthologs of *H. contortus*. We chose the four most genetically related orthologs as a base to examine for variation, but as stated by both the reviewer and us, more broad examination is needed. We have amended the below sentence to better make this point.

“In addition, as noted among *Caenorhabditis* species, we examined a single amino acid site in four *H. contortus* cytochrome P450s, and alteration of TBZ responses could be caused by variation at other sites or in other cytochrome P450s.” **Line 2: "independent" should read "independently" (since it is an adverb modifying the verb 'modifies').**

We made this edit. **Line 55: "identified by another mapping technique" might be better written more specifically; maybe it could be something like "identified by linkage and fine mapping"?**

We have revised to say “identified by linkage and fine mapping”. **Line 75: "in genes independent of beta-tubulin" might be better written as "in additional genes other than beta-tubulin".**

We have modified the sentence as recommended to “in additional genes other than beta-tubulin.”

**Lines 99-100: "Using the cycle of discovery" might be better written as "Using this cycle of discovery".**

We have made the suggested change to “Using this cycle of discovery…”

**Line 105: "populations and research" might be better written as "populations, and research".**

We have added the comma.

**Line 106: "independent" should read "independently".**

Modified

**Lines 118-119: Might "and was significantly favored" be better written as "but was significantly favored"?**

We have modified the “and” to “but”.

**Figure 1A: The label on the Y-axis ("TBZ Response") is confusing. Would it be acceptable to relabel it as "TBZ Resistance", which is what I think it really shows?**

As noted above, we have made the suggested change.  **Line 191: What is an "Eigen significance threshold"? How is it defined? [This question also arises at Line 980 of the Supplementary Figures.]**

We have better clarified the EIGEN thresholding in the methods: “Significance thresholds were determined using Bonferroni and Eigen significance values. The Bonferroni threshold is based on the number of markers in the analysis, and the Eigen threshold corrects for the number of genetically independent markers in the data set. The Eigen threshold takes advantage of the extensive linkage disequilibrium in *C. elegans* to limit the number of “unique” markers [(Zdraljevic et al. 2019)](https://paperpile.com/c/r0ttsh/zdRW).”

**Figure 2 and Line 229: There is inconsistent labeling of a lower significance threshold; it is labeled with two asterisks ('**') in the figure itself, but apparently with three asterisks ('***') in the figure legend. This inconsistency should be corrected.**

We have corrected the figure legend.

**Figure 4 and Lines 297-298: A single asterisk ('*') is labeled as being p < 0.05, but there is no explanation given for what four asterisks ('****') mean; this should be corrected.**

As noted above, we have made this correction.

**Figure 5 and Lines 310-311: A single asterisk ('*') is labeled as being p < 0.05, but does not exist anywhere in the figure; the figure does have four asterisks ('****'), but there is no explanation given for what they mean. This should be corrected.**

As noted above, we have made this correction.

**Line 392: "the cytochrome P450" might better read "a cytochrome P450".**

Revised as recommended.

**Line 393: "independent" should read "independently".**

Revised as recommended.

**Line 704: "Haemoncus" should read "Haemonchus".**

Edited. **Line 980: Again, what is an Eigen threshold and how is it defined?**

See above for line 191.

**Figure S7 and Lines 1075-1076: There is a significance of one asterisk ('*') shown in the figure, but not defined in the figure legend; this should be defined.**

As noted above, we have made this correction.

**Line 1096: "Figure S9" was corrected but not fully corrected!**

We have removed the three asterisk description in the legend.

*Reviewer 3:*

**• Metabolites. It is surprising that there are not cascading effects of the allele across all metabolites in this pathway. That is, if the allele impacts the concentration of a metabolite upstream, then one would naively expect downstream metabolite concentrations to also be impacted. Also, wouldn't the K267E substitution be predicted to increase, not decrease TBZ-OH abundances? (I may be wrong about that) Anyway, it is suggested that "additional detoxification" mechanisms are likely to be at play, but could it at all be speculated in the discussion what might be going on here?**

As noted above, because of differences in metabolic flux, comparisons in metabolomics and analysis of the cascading effects of the variant on metabolism are difficult to understand. We focused on a simplified explanation of the metabolic effects for which the conclusions are based on obvious interpretations of the presented data. The enzyme with the lysine allele is the more active version, and we expect that the metabolism of the K267E allele would be slower, which is represented by the significant difference shown for TBZ-OH in Figure 4.

**• Fitness, trade-offs, and cytochrome P450. For me, cytochromes are implicated in the electron transport chain and thus might be expected to have wide-ranging impacts on metabolism. Little did I realize that these proteins are also often implicated in xenobiotic metabolism specifically. While this is mentioned in the introduction, I think fleshing this out a little bit more in the discussion may be warranted. Mainly because, without this context, the lack of fitness trade-offs connected to this allele remain unexplained. That is, if CYP-35D1 were expected to be pleiotropic, one might expect the lysine allele to be at a disadvantage in the absence of thiabendazole. Along the same lines-- how many CYP proteins are present in the C. elegans genome? If there is a lot of copy number variation, then the opportunity for redundancy and neofunctionalization is high, which would also help to explain the apparent lack of a trade-off here. And an additional minor point, I was surprised that so few Caenorhabditis species were included in the phylogenetic analysis of CYP-35D1 in Figure 7 as there are many genomes now available (maybe there are species out there with the 267 lysine?). Alternatively, I might have missed that this gene only has single-copy orthologs in the reported species; in which case, a larger gene family tree might better contextualize this result.**

Although the points presented are interesting and relevant, conclusions from available data are difficult to make because many pathways involving cytochromes are not characterized and the role for each cytochrome in xenobiotic metabolism is unclear. In addition, we chose a representative set of species across the *Caenorhabditis* genus so as not to generate an increasingly crowded tree.

**Data and code availability. While it is claimed that the data and code have been made available (https://github.com/AndersenLab/2023_cyp35d1_TBZmanuscript), this link leads to a 404 error message. I also did not find any NCBI accession/bioproject numbers associated with this work. I do appreciate the extensive sharing of data in the supplemental materials.**

The 404 error was caused by a permissions issue with the GitHub repo. The repo has now been made public. Also the repo has been renamed to “2024_cyp35D1_TBZ_manuscript”.

**Figure 1A. This figure might be clearer if, in the legend or the figure itself, it is noted that body length is being used as the proxy for "response" depicted here.**

Response was an error in the figure and has been revised to indicate that what is being shown is the level of resistance.

**Line 158. The Andersen et al. 2015 paper should be cited here to clarify that these RILs were previously constructed.**

Cited.

**Lines 473-480. Were the guide RNA sequences reported in these cited papers? Or, I may have missed these somewhere.**

As above, we have modified S File 18 to include the oligonucleotide sequences.
